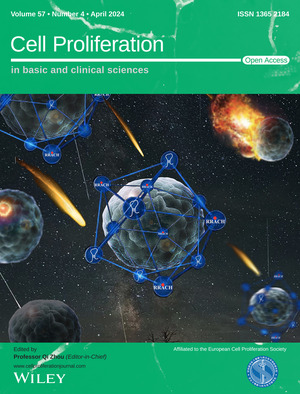# Featured Cover

**DOI:** 10.1111/cpr.13642

**Published:** 2024-04-01

**Authors:** Yizhou Jin, Zhipeng Fan

## Abstract

The cover image is based on the Review *New insights into the interaction between m6A modification and lncRNA in cancer drug resistance* by Yizhou Jin and Zhipeng Fan  https://doi.org/10.1111/cpr.13578.